# Shortening duration of untreated illness in young people with first episode eating disorders: protocol of a randomised controlled feasibility trial of a smartphone friendly multi-modal decision-making tool (FREED-M) to improve help-seeking

**DOI:** 10.1186/s40814-024-01585-2

**Published:** 2025-02-07

**Authors:** Luiza Grycuk, Dina Monssen, Molly R. Davies, Helen Sharpe, Karina L. Allen, Vibhore Prasad, Rachel Potterton, Priya Popat, Victoria A. Mountford, Sheryllin McNeil, Vanessa Lawrence, Nina Grant, Kimberley A. Goldsmith, Danielle Glennon, Sarah Byford, Amy Brown, Ulrike Schmidt

**Affiliations:** 1 Centre for Research in Eating and Weight Disorders (CREW), Department of Psychological Medicine, Institute of Psychiatry, Psychology & Neuroscience, King’s College London, London, UK; 2 Department of Clinical and Health Psychology, School of Health in Social Science, University of Edinburgh, Edinburgh, UK; 3 The Eating Disorders Outpatient Service, Maudsley Hospital, South London and Maudsley NHS Foundation Trust, London, UK; 4 School of Population Sciences, King’s College London, London, UK; 5 School of Medicine/Lifespan and Population Health, University of Nottingham, Nottingham, UK; 6 Specialist Eating Disorder Service, Forward Thinking Birmingham, Birmingham Women’s and Children’s Foundation Trust, Birmingham, UK; 7 King’s Health Economics, Institute of Psychiatry, Psychology & Neuroscience, King’s College London, London, UK; 8 Maudsley Centre for Child and Adolescent Eating Disorders, South London & Maudsley NHS Foundation Trust, London, UK; 9 Department of Biostatistics and Health Informatics, Institute of Psychiatry, Psychology & Neuroscience, King’s College London, London, UK; 10 Sussex Eating Disorder Service, Sussex Partnership NHS Foundation Trust, Worthing, UK

**Keywords:** Eating disorders, Anorexia nervosa, Bulimia nervosa, Binge eating disorder, Early intervention, Feasibility trial, Online intervention

## Abstract

**Background:**

Early intervention gives young people the best chance to recover from eating disorders (EDs). An important focus of early intervention is shortening the time between a person first developing symptoms and starting treatment (duration of untreated eating disorder; DUED). Patient-related factors (e.g. poor mental health literacy and help-seeking difficulties) are strongly associated with DUED. The aims of our study are to co-design and test the feasibility of FREED-Mobile (FREED-M), an online intervention tool for young people with early-stage EDs. This tool aims to improve knowledge about EDs, increase motivation to seek treatment and teach early steps towards change or recovery, thus reducing DUED.

**Methods:**

We will carry out a randomised controlled feasibility trial comparing the FREED-M tool with a control intervention where individuals are sign-posted to an ED charity website. The objectives of the proposed trial are to establish/estimate: (a) attrition rates at follow-up (primary feasibility outcome); (b) participant recruitment; (c) intervention uptake, completion rates and acceptability; (d) intervention effect sizes and standard deviations for outcomes to inform the sample size calculation for a large-scale randomised controlled trial (RCT); (e) stakeholder views on the intervention.

We aim to recruit 116 participants (young people, aged 16–25, with first episode ED) from primary care, schools and universities, ED services and social media. Online assessments will be carried out at baseline, end of intervention and follow-up (weeks 0, 4 and 12 post-randomisation, respectively). Outcomes will include motivation and readiness to change, attitudes and intentions towards help-seeking, ED symptoms, mood and social functioning, and health-related quality of life. Additionally, we will carry out a qualitative evaluation of participants’ views of the intervention and study design.

**Discussion:**

The results of this feasibility trial will inform adaptations to the intervention as needed, as well as the study design (e.g. sample size, primary outcomes) of a future large-scale RCT to assess the effectiveness of the FREED-M intervention. If effective, this novel, online intervention has the potential for wide dissemination and for substantially reducing DUED to improve long-term patient outcomes.

**Trial registration:**

ISRCTN, ISRCTN15662055. Registered 27 July 2022, https://www.isrctn.com/ISRCTN15662055.

**Supplementary Information:**

The online version contains supplementary material available at 10.1186/s40814-024-01585-2.

## Introduction

Eating disorders (EDs; anorexia nervosa, bulimia nervosa, binge eating disorder and related partial or mixed syndromes) are highly distinctive disorders at the brain-body interface. They affect up to 15% of young women and up to 5.5% of young men in Western countries [1, 2]. The COVID-19 pandemic saw an increase in prevalence, demand for healthcare services and symptom severity of EDs [3–7]. The peak age of onset of EDs is from adolescence into emerging adulthood (age 15–25 years) with a median of 18 years, considered to be a developmentally sensitive time [8, 9]. The average illness duration is ~ 6 years [10]. Comorbidities, with ensuing long-term physical and psychosocial disability, are common in all EDs [11]. Compared to the general population, mortality rates for EDs are almost doubled and ~ 6 times higher for people with anorexia nervosa, which is higher than any other psychiatric disorder [12]. Estimates of the economic and social impact of EDs suggest a disease burden that is comparable to anxiety and depression [1].

It is critical to intervene early in the first episode of an eating disorder since this is when symptoms are most malleable and to minimise the negative impacts of illness on young people’s development. Longer illness duration is one predictor of poor outcomes in EDs [13]. Other predictors include high levels of perfectionism and dieting/disordered eating in adolescence [14]. The repeated reinforcement of behaviours associated with the ED over time makes these more entrenched and habitual [15]. Similar to psychosis and bipolar disorder [16, 17], converging data support the idea that neurobiological changes associated with disordered eating may alter the illness trajectory of EDs [15, 18]. Evidence suggests that early-stage ED can be defined as < 3 years of illness duration, which is when treatment responses appear most favourable. Beyond this time frame, treatment response is significantly poorer [19]. In a recent systematic review of 14 studies of the duration of an untreated ED (DUED) in first-episode EDs [20], we found an average DUED of 29.9 months for anorexia nervosa, 53.0 months for bulimia nervosa and 67.4 months for binge eating disorder [21]. This suggests that many young people accessing treatment have been unwell for 3 or more years. Of note, emerging adults (age 18–25) with EDs on average have a DUED which is approximately 50% longer than that of adolescents below age 18 [22]. This may be due to greater help-seeking barriers; for example, being independent from their families and having to make their own decisions about their health care, or transitioning from child to adult mental health services [23, 24]. Those in certain groups such as minoritised ethnic groups, the LGBTQ + population, gender diverse individuals, those with high body mass index (BMI) and males have additional barriers to overcome when seeking help and treatment [25, 26].

A large United Kingdom (UK) survey of people with an ED found that it took, on average, 21 months from the onset of symptoms to the person realising they had a problem and another 13 months before they consulted their general practitioner (GP) [21]. Another study conducted in Australia found an average delay of 5.3 years between the onset of ED symptoms and seeking treatment [27]. DUED is thought to have three broad stages: firstly, a period where people do not recognise they have a problem; secondly, a period where they recognise they have a problem but are not ready to seek help or do not know how to seek help; thirdly, a period where they have sought help and are awaiting treatment. Whilst the first two components are patient-related, the third encompasses service-related delays [21, 28].

To shorten service-related delays, we developed an early intervention service model and care pathway for young people (age 16–25) with early-stage EDs called First Episode Rapid Early Intervention for EDs (FREED) [29]. The FREED model is biopsychosocial, with a focus on optimising early care. A single-centre pilot evaluation found significantly improved waiting times, DUED and clinical outcomes in FREED patients compared to a cohort of patients of similar age and illness duration seen previously in the same service [30, 31]. The introduction of FREED reduced DUED by about 6 months when delivered as intended (without external barriers to access, e.g. delays due to lack of funding or patient-related scheduling issues) [32]. At 12 months post-treatment, ~ 60% of FREED anorexia nervosa patients returned to normal weight (BMI of 18.5 kgs/m^2^ or above) versus only ~ 17% of similar patients seen in the same service 2 years before FREED was implemented [31]. These differences in recovery rates persisted at 24 months [33]. The introduction of FREED services also reduced the need for more intensive treatment (inpatient, day patient) by ~ 36%, compared to treatment as usual [31].

In 2016, FREED was introduced to three other large ED services in England. Data from this multi-center study showed similar reductions in DUED as our pilot study [32]. Since 2017, this model has expanded to other services across England (FREED-4-All) and early evaluation provided preliminary evidence for the acceptability and effectiveness of the model at scale [34]. In addition, FREED was included in National Health Service (NHS) England commissioning guidance for adult ED services as a best practice example and adopted for national roll-out in England [35]. As of January 2023, 53 out of 54 Mental Health Trusts in England had adopted FREED.

Studies from England and other Western countries suggest that 10–32% of ED patients referred by their GP for a specialist assessment do not attend [36–38], and a further 27% of those who do attend the assessment do not take up treatment [36, 39]. Patient-related components of DUED and their impact on treatment therefore need to be addressed. These patient-related barriers to treatment include (a) *attitudinal* (shame, self-stigmatisation as undeserving, negative attitudes towards seeking help) [1, 40–42], (b) *knowledge-based* (inability to recognise the severity of the illness, lack of knowledge about available help) [41, 43] and (c) *motivational* (ambivalence about change) [42, 44] barriers.

Theory-based interventions to improve knowledge, beliefs and motivation are needed to improve help-seeking behaviours and reduce poor treatment uptake and engagement in EDs. A tool for designing behaviour change interventions is the Behaviour Change Wheel, a framework that identifies three essential conditions required to enable behaviour change to occur: capability, motivation and opportunity [45, 46]. Applying this framework to EDs, patient-related barriers to seeking help and engaging with treatment relate to limited psychological capability (e.g. poor knowledge about symptoms) [47], as well as limited motivation to change [21, 22]. Psychological capability can be improved through increased knowledge, understanding, or training in emotional, cognitive and/or behavioural skills. Motivation can be improved through increasing knowledge and understanding and altering feelings about a behavioural target [45, 46].

Psychoeducation refers to education offered to individuals with a mental health problem and their families to help them manage their condition. Recently, psychoeducation has emphasised EDs as brain-based and biological rather than sociocultural disorders. Whilst a focus on biogenetic causes may reduce parents’ self-blame and stigmatisation of EDs [48], there is growing evidence across a multitude of disorders, including depression [49–51], anxiety [52], attention deficit hyperactivity disorder [53] and EDs [54], that biogenetic illness explanations may reduce patients’ hope of recovery and self-efficacy of being able to change. Instead, psychoeducation that emphasises biological malleability, i.e. reversible changes to brain, body and behaviour, instils hope, optimism and self-efficacy regarding recovery [49, 50, 54], may be more effective in ensuring early help-seeking and engagement with services.

Delivery of education through multimedia messages, rather than textual information alone, facilitates more in-depth information processing, the development of new perspectives and self-discovery [55]. It also increases satisfaction and emotional connection with the material, especially if the audio-visual component includes a personal element (e.g. telling a story) through film or animation [55–57]. As real-life images can fatigue or desensitise the viewer when they have previously been exposed to similar images, the animation may provide more engaging visual materials for health education [58–61] and advocacy (e.g. United Nations Children’s Fund’s [UNICEF’s] Smurf video illustrating the impact of war on children [https://www.youtube.com/watch?v=bftmZlpAkPg]) [62].

Feedback is the provision of verbal, written, or graphically displayed information to a person about their behaviour, health, or risk of ill health, based on their personal characteristics. Most models on the efficacy of feedback focus on the motivational and learning aspects of personalised feedback, e.g. feedback increases the likelihood or depth of processing information or modifies knowledge, beliefs, or behaviour [63]. Results from meta-analyses indicate that feedback has a significant positive effect on psychotherapy outcome [64, 65]. The few studies which used personal feedback about symptoms and progress as part of ED interventions have had promising results [66–72].

To address these important gaps in access to care and treatment, our proposed multi-media decision-making intervention for first-episode EDs (FREED-Mobile [FREED-M]; https://freedm.uk/) will address key issues raised above, e.g. via improving help-seeking and treatment motivation and will seek to ultimately reduce the patient-related component of DUED in young people with EDs. To the best of our knowledge, there are no other comparable online interventions, apps, or decision-making tools available.

### Study aims and progression to full trial

#### Our aims are twofold

Firstly, we aim to develop a smartphone-friendly multi-modal online decision-making tool (https://freedm.uk/) for young people with a first episode ED to increase motivation for treatment and change, help-seeking from primary care and attendance at specialist assessment/treatment and thus reduce DUED. Specifically, the intervention aims to increase motivation to change/seek treatment through psychoeducation and personalised feedback on symptoms and highlighting steps towards seeking and getting help.

Secondly, we aim to carry out a feasibility randomised controlled trial (RCT) comparing the intervention to a credible control intervention. The objectives of the proposed trial are to establish/estimate: (a) attrition rates at follow-up (primary feasibility outcome); (b) participant recruitment from different sources; (c) intervention uptake, completion rates and acceptability; (d) intervention effect sizes and standard deviations for outcomes to inform the sample size calculation for a large-scale RCT; (e) stakeholder views on the intervention, optimal delivery pathways and study procedures.

#### Progression to full trial

Key indicators of study success and progression will be: (a) ability to recruit and (b) retain participants in the study (primary feasibility outcome). Progression from feasibility to a future definitive RCT would be warranted based on the criteria detailed below:

- Go: recruitment as planned (total trial *N* = 116, i.e. 58 participants per arm) and < 40% attrition (attrition = no follow-up assessment).
- Stop: recruitment below 70% of planned and/or attrition > 50%.

If recruitment and retention are between stop-and-go criteria (i.e. recruitment between 70 and 100% of planned and attrition between 40 and 50%): solid remedial action would need to be taken for the study to progress to a full RCT. This could include additional sites/recruitment methods or alterations to the intervention, based on qualitative feedback.

## Methods and analysis

This protocol paper follows the guidelines of Standard Protocol Items: Recommendations for Interventional Trials (SPIRIT) [73] and the guidance given by Thabane and Lancaster [74].

### Study design

**Fig. 1:**
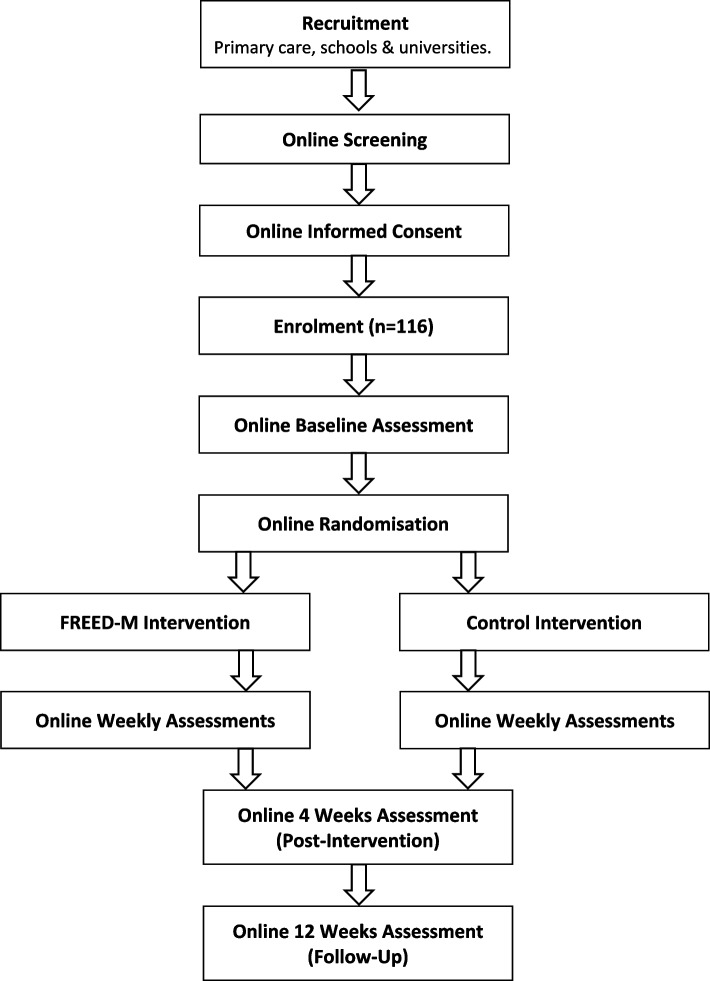
Trial flowchart

This is a parallel group, two-arm feasibility trial. Participants will be randomly allocated to either FREED-M intervention or sign-posted to the resources on a reputable ED charity website. Participants will be recruited from primary care, schools and universities across the catchment areas of participating services, through FREED services and from social media using geotargeting. Outcomes will be measured at baseline (week 0), post-intervention (4 weeks post-randomisation) and follow-up (12 weeks post-randomisation). Selected clinical outcomes assessing ED symptomatology will additionally be measured at weeks 1–3. Figure 1 and Table 1 give details of all assessments and time points.

Figure 1 details the participant flow through the study. The timings for the post-intervention and follow-up assessments are given as time post-randomisation (i.e. 4 weeks post-randomisation and 12 weeks post-randomisation, respectively). *Abbreviations:* *n *= number of participants; FREED-M = first episode rapid early intervention for eating disorders

**Table 1:**
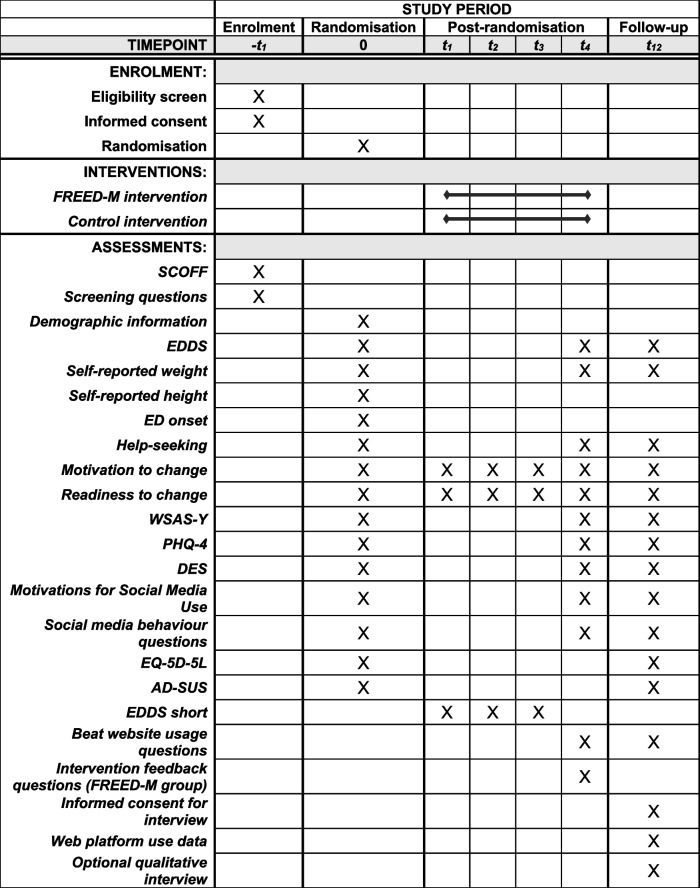
Trial assessments and timepoints

Table 1 displays the frequency of questionnaires, scales and tasks, and timepoints administered

*Abbreviations*: *t *timepoint, *SCOFF* 5-item eating disorder screener, *EDDS *Eating Disorder Diagnostic Scale, *ED *eating disorder, *WSAS-Y* Work and Social Adjustment Scale–Youth, *PHQ-4 *Patient Health Questionnaire 4 questions, *DES *Disclosure Expectations Scale, *EQ-5D-5L *EuroQol health-related quality of life measure, *AD-SUS *Adult Service Use Schedule

### Participants

Inclusion criteria entail: young people, aged 16–25, with a suspected ED, defined by a score of 2 or above on a widely used screening instrument for EDs (the ‘SCOFF’) [75]. Exclusion criteria entail: people with current or past specialist treatment for their ED, those who cannot understand spoken and written English, or who do not have access to the Internet.

### Sample size

We have chosen a sample size of *n* = 35 per group, which is at the upper end recommended for feasibility trials [76]. We assume an attrition to follow-up rate (a) of 0.4. This is based on data from our ongoing European Union-consortium of internet-based prevention and treatment of common mental disorders, including EDs. Applying an attrition correction factor of 1/(1-a), we will need a sample size of *n* = 116, i.e. 58 participants/group. Assuming 116 eligible participants enrol, we will be able to estimate a 40% attrition rate within a 95% confidence interval of ± 9%.

### Randomisation and blinding

After the baseline assessment, participants will be randomly allocated to either the FREED-M intervention or signposted to the resources on the ED charity website (control group). A random allocation list will be created and maintained by the King’s Clinical Trials Unit using an online system to ensure allocation concealment. Minimisation will be used to balance groups for prognostic factors (anorexia nervosa vs other ED type; specialist referral in progress [yes/no]). Due to the nature of the study, neither participants nor the research team will be blinded to participant condition assignment. However, care will be taken to present interventions in both trial arms as equivalent to reduce participant expectancy biases.

### Recruitment

Participants will be recruited from primary care, schools and universities directly, and via online advertising on social media (e.g. Facebook®, Instagram®) using geotargeting, across the catchment areas of the FREED Network, which currently includes adult ED services in 53 out of the 54 eligible Mental Health Trusts across England. These services are trained in and signed up to deliver early intervention for EDs in emerging adults. These areas will also all have Child and Adolescent ED teams with an early intervention ethos. The reason for conducting the study in areas with existing early intervention services is (a) to ensure that if young people are mobilised to seek help early, there are services in their area that deliver this and (b) to reduce variability in service-related aspects of DUED in participants.

### Consent

Participants will provide consent online via the FREED-M website. In schools with further regulations, schools may wish to notify parents about the project and obtain parental (carer) approval, according to their own policies (e.g. privacy policies). Schools will be asked to use parental ‘opt out’ rather than ‘opt in’, so that the threshold for young people wishing to join the study remains low. EDs can be experienced as a shameful secret, and it is therefore important that young people can access help and support without their parents’ knowledge [77].

### Assessment procedures

Online screening, consent and baseline assessment will be carried out before randomisation. Following the baseline assessment (week 0), eligible participants will be randomly allocated to either the FREED-M intervention or the control group. Participants in both groups will then complete three brief assessments (weeks 1–3), a post-intervention assessment (4 weeks post-randomisation) and a follow-up assessment (12 weeks post-randomisation); all of these will be completed online.

### Outcome measures

#### Feasibility outcomes

1. Primary feasibility outcome: attrition rates at follow-up (the proportion of randomised participants who did not complete the follow-up assessment)
2. Other feasibility outcomes: participant recruitment (proportions of randomised participants recruited from different sources; number of participants recruited per month); completion rates (proportion of randomised participants who completed all assessments); intervention uptake (proportion of randomised participants who accessed at least a part of the first intervention module); intervention completion rates (proportion of randomised participants who accessed each intervention module); intervention acceptability (proportion of participants in the intervention group with a rating of ≥ 4 on each feedback question, scored on a five-point Likert scale from 1 = did not like it at all/ not at all useful to 5 = best thing ever/extremely useful).

#### Clinical outcomes

1. ED-related outcomes: at baseline only, participants will complete a questionnaire on ED symptom onset abbreviated from a comprehensive onset interview from our early intervention studies [30–32] and report their height. At all assessment points (baseline, weeks 4 and 12) they will complete the Eating Disorder Diagnostic Scale (EDDS) [78], self-reported weight, questions on motivation and readiness to change [79] and questions on ED help-seeking. A short version of the EDDS and the questions on motivation and readiness to change will additionally be completed in weeks 1–3.
2. Other clinical outcomes: questionnaires assessing depression and anxiety (PHQ-4) [80, 81], Disclosure Expectations Scale (DES) [82], social media beliefs and motivation for use (Social Media Use [83], Motivations for Social Media Use Scale [84]) and social impairment and functioning (WSAS-Y) [85] will be completed at baseline, weeks 4 and 12.

#### Health-related quality of life outcomes

The adult, five-level version of the EuroQol measure (EQ-5D-5L) will be completed at baseline and 12-week follow-up to assess health-related quality of life (HRQoL). This version of the EQ-5D was selected because it was introduced to improve the sensitivity of the measure [86] and, despite being a measure for adults, is recommended by the EuroQol group for participants aged 16 and older [87]. Given the age range in the current study, we will assess the acceptability of this measure for our study participants.

#### Intervention/service-related outcomes

1. A modified, self-report version of the Adult Service Use Schedule (AD-SUS) will be tested for acceptability and comprehensiveness at baseline and week 12, in line with previous versions developed for application to both children and young people and young adults [88]. The measure was adapted based on existing evidence of service use in EDs [89, 90]. Service use will be collected from the NHS and social services perspective preferred by the National Institute for Health and Care Excellence (NICE), including education-based health and social services. The feasibility of ascertaining NHS secondary care service use information from electronic patient records at participating ED services will also be assessed.
2. Questions regarding the use of resources from the ED charity website will be asked to both groups at week 4 and 12, and intervention feedback questions will be asked to the intervention group only at week 4.

### Intervention

The FREED-M intervention consists of an integrated series of four short, animated films focusing on key psychoeducational messages around help-seeking (identifying and seeking help for symptoms), the body (highlighting gut health), social media (effects of and strategies for managing social media including diet/health/fitness) and the malleability of changes to cognition (e.g. thinking style, socioemotional processing, relationships). Animations are supplemented by downloadable in-depth online information that participants can access. The psychoeducation topics and intervention content were selected and developed in co-production with young people who have a lived experience of an ED and experts in the field, including both researchers and clinicians. The content for the animations was further informed by prior qualitative research. For example, we previously identified barriers to help-seeking specific to emerging adulthood [23]. Individual stories and quotes from this research study were included in the help-seeking animation. This process was informed by recent guidance on the development of electronic and mobile health tools [91, 92]. The online FREED-M intervention was developed to be smartphone-friendly but is delivered via a website and can be accessed via a mobile phone or desktop computer.

Participants allocated to the FREED-M intervention will receive personalised feedback about their symptoms and behaviours, including information on medical risk mitigation and advice on behaviour change and help-seeking. They will be able to access one animation per week and following each animation they will be invited to briefly reflect on the content and answer questions on the personal relevance of the materials shown. They will also be able to access weekly downloadable resources.

Participants will be able to download their feedback data and share it with their GP. This may be particularly useful in cases where the young person first needs to approach their GP to start their help-seeking process.

#### Control intervention

Participants allocated to the control intervention will be signposted to the website of ‘Beat’ [21], a reputable UK ED charity which provides information, resources and support for eating disorders. Participants in this group will be able to access the FREED-M intervention after the end of the trial.

### Interviews

An estimated sample of around 20 participants, depending on inductive thematic saturation [93], will take part in qualitative interviews about their experience of the intervention. Interviews will be based on a specifically designed semi-structured topic guide (see Appendix 1) trying to comprehensively capture people’s views and will explore attitudes towards the recruitment methods and study design, initial expectations of the intervention, perceived strengths and weaknesses, engagement and suggestions for enhancing acceptability and relevance. Participants will be recruited purposively from the intervention group, e.g. according to gender, age and baseline ED symptoms, to capture a broad range of participants with potentially different experiences. Participants will be approached for these interviews at the 12-week post-randomisation time point, to ensure that they have had the chance to complete the intervention.

### Data analysis

#### Feasibility/clinical data

Outcomes, including feasibility outcomes, will be summarised as mean and standard deviation, median and interquartile range, frequency and proportion, or count and Poisson confidence interval, as appropriate. FREED-M intervention engagement metrics will be summarised for the intervention arm only. The potential outcomes for a future trial will be summarised appropriately overall and in each group. Completeness and variability of the outcome measures will be determined and used together as a guide to which might be best utilized as a primary outcome in a future definitive trial. As this is a feasibility trial, no formal statistical tests of differences between the groups will be conducted. Summary estimates will be used in calculating the sample size of the future large-scale RCT. All analyses will be conducted using STATA v14.

#### Economic data

The acceptability of the draft AD-SUS and the HRQoL measure (EQ-5D-5L) will be explored using completion rates (proportion of randomised participants who completed the AD-SUS or EQ-5D-5L, respectively). The comprehensiveness of the draft AD-SUS will be assessed through the identification of redundant items (services included in the AD-SUS with little or no use reported by participants) and missing items (services not included in the AD-SUS but reported by participants under ‘other’). This data will be used to produce a modified version of the AD-SUS suitable for a subsequent RCT. Service use and HRQoL will be summarised and reported descriptively (mean, SD, ranges, etc.).

#### Qualitative data

We will use framework analysis to facilitate analysis both within and across young people [94–96]. The interpretivist assumptions underpinning our approach emphasise the importance of understanding individuals’ experiences and interpretations from their points of view. No specific theory will be used to guide the development of the coding framework. However, given the semi-structured nature of the topic guide, this is likely to influence the generation of the initial framework used to code the interviews. In short, transcripts will be reread to ensure data familiarisation. Two researchers will independently code a minimum of five transcripts and discuss alternative viewpoints before reaching a consensus on a provisional analytical framework to be applied to subsequent transcripts. An iterative method, in which the data extracts are continuously checked for coherence and the coding framework refined, will be followed. Data will be summarised through charts to map the range of views and experiences and to allow the wider research team to engage with and offer interpretations of the data.

### Patient and public involvement

In preparation for the first stage of the protocol development, we conducted a focus group with five young adult ED patients to identify topics for psychoeducation and how this should be delivered. Three experts by experience reviewed different proposal stages. The animations and resources included in the intervention were developed iteratively in consultation with ED clinicians and young people in our target age range, current patients and/or those with current or prior lived experience. Two patients/experts by experience will be part of the trial steering group. Jointly with the core research team, they will: be involved in aspects of study design, oversee the running of the study, be involved in designing a future large-scale RCT by appraising findings from the feasibility study, and contribute to dissemination. In addition, other young people will be involved in beta-testing and dissemination on an ad hoc basis. These will be recruited from our volunteer registry of FREED patients (FREEDom Finders) and via social media. Participation will be balanced in terms of gender, age and ethnicity. Patient consultants to the project will be remunerated in line with INVOLVE guidance [97].

## Ethics and dissemination

### Ethics and safety consideration

This trial will be conducted in compliance with the Declaration of Helsinki, the principles of good clinical practice (ICH-E6 guideline), and the ICH-E8 and E9 guidelines, and in accordance with all applicable regulatory requirements including but not limited to the UK policy framework for health and social care research. The results of this trial will be reported in accordance with the CONSORT 2010 Statement [98]. This trial is registered in the ISRCTN register (ISRCTN15662055). Ethical approval was obtained from the London-Camden & Kings Cross Research Ethics Committee (REC Ref: 22/LO/0655) on 11th October 2022.

We are aware that some young people joining the trial may experience significant distress and/or suicidal symptoms. Irrespective of which group they are allocated to, each page of the intervention will display a clickable button that reads ‘I need urgent help’. Clicking this button will signpost them to the Young Minds urgent help page (https://www.youngminds.org.uk/young-person/find-help/i-need-urgent-help/), which provides details of confidential support and crisis hotlines for young people in distress or at risk of suicide.

### Dissemination plan

This novel intervention has the potential to substantially improve help-seeking and ultimately reduce DUED. If successful, and after carrying out a future large-scale trial, we would disseminate the intervention widely in NHS (including both primary and secondary care), community and education settings.

We will disseminate our findings to young people and families via relevant organisations. This will include conference presentations and information on the websites of these organisations and other relevant websites (such as FREEDfromED.co.uk, EDIFYresearch.co.uk, and the King’s College London websites). To reach young people, we will post information on social media feeds (e.g. Instagram, Facebook and Twitter®) of relevant organisations and selected influencers. Finally, we will work with our press office to disseminate the work to the press and other media outlets.

We will disseminate our findings to schools and universities via relevant organisations/conferences (e.g. Personal, Social, Health and Economic Education (PSHE) teachers’ organisation, schools in Mind Network) and student mental health and well-being organisations.

We will disseminate study findings to health professionals via scientific papers and relevant conferences such as large UK and international primary care, early intervention and ED conferences. We will work with Academic Health Sciences Networks (AHSNs) to facilitate the adoption and spread of our FREED-M innovation across all areas of healthcare provision. We will work towards getting our intervention adopted into NHS Mental Health online resource libraries and endorsed/recommended by relevant national and international guidance documents/organisations.

### Trial progress

Participant recruitment and data collection for this study began in November 2022. Amendments to the study protocol will be reported in publications reporting the study outcomes.

## Supplementary Information

Supplementary Material 1.

## Data Availability

Not applicable.

## Abbreviations

AD-SUS: Adult service use schedule
AHSN: Academic Health Sciences Networks
BMI: Body mass index
DES: Disclosure expectations scale
DUED: Duration of an untreated eating disorder
ED: Eating disorder
EDDS: Eating Disorder Diagnostic Scale
GP: General practitioner
HRQoL: Health-related quality of life
FREED: First Episode Rapid Early Intervention for Eating Disorders
FREED-M: First Episode Rapid Early Intervention for Eating Disorders-Mobile
LGBTQ +: Lesbian, gay, bisexual, transgender, queer/questioning and others
NHS: National Health Service
NICE: National Institute for Health and Care Excellence
PHQ: Patient health questionnaire
PSHE: Personal, Social, Health and Economic Education
RCT: Randomised controlled trial
UK: United Kingdom
UNICEF: United Nations Children’s Fund
WSAS: Work and Social Adjustment Scale

## References

[1] Schmidt U, Adan R, Böhm I, Campbell IC, Dingemans A, Ehrlich S, et al. Eating disorders: the big issue. The Lancet Psychiatry. 2016;3(4):313–5. 27063378 10.1016/S2215-0366(16)00081-X

[2] Limbers CA, Cohen LA, Gray BA. Eating disorders in adolescent and young adult males: prevalence, diagnosis, and treatment strategies. Adolesc Health Med Ther. 2018;9:111–6. 30127650 10.2147/AHMT.S147480 PMC6091251

[3] Taquet M, Geddes JR, Luciano S, Harrison PJ. Incidence and outcomes of eating disorders during the COVID-19 pandemic. Br J Psychiatry. 2022;220(5):262–4. 10.1192/bjp.2021.105 PMC7612698 35048812

[4] Linardon J, Messer M, Rodgers RF, Fuller-Tyszkiewicz M. A systematic scoping review of research on COVID-19 impacts on eating disorders: a critical appraisal of the evidence and recommendations for the field. Int J Eat Disord. 2022;55(1):3–38. 34773665 10.1002/eat.23640 PMC8646470

[5] Shaw H, Robertson S, Ranceva N. What was the impact of a global pandemic (COVID-19) lockdown period on experiences within an eating disorder service? A service evaluation of the views of patients, parents/carers and staff. J Eat Disord. 2021;9(1):14. 33468242 10.1186/s40337-021-00368-x PMC7814524

[6] Zipfel S, Schmidt U, Giel KE. The hidden burden of eating disorders during the COVID-19 pandemic. The Lancet Psychiatry. 2022;9(1):9–11. 34921799 10.1016/S2215-0366(21)00435-1 PMC8673860

[7] Haripersad YV, Kannegiesser-Bailey M, Morton K, Skeldon S, Shipton N, Edwards K, et al. Outbreak of anorexia nervosa admissions during the COVID-19 pandemic. Archives of Disease in Childhood. 2021;106(3):e15-e. 10.1136/archdischild-2020-319868 32709684

[8] Micali N, Hagberg KW, Petersen I, Treasure JL. The incidence of eating disorders in the UK in 2000–2009: findings from the General Practice Research Database. BMJ Open. 2013;3(5):e002646. 10.1136/bmjopen-2013-002646. 10.1136/bmjopen-2013-002646 PMC3657659 23793681

[9] Solmi M, Radua J, Olivola M, Croce E, Soardo L, Salazar de Pablo G, et al. Age at onset of mental disorders worldwide: large-scale meta-analysis of 192 epidemiological studies. Mol Psychiatry. 2022;27(1):281–95. 10.1038/s41380-021-01161-7 PMC8960395 34079068

[10] Royal College of Psychiatrists. Position statement on early intervention for eating disorders. 2019. Available from: https://www.rcpsych.ac.uk/docs/default-source/improving-care/better-mh-policy/position-statements/ps03_19.pdf?sfvrsn=b1283556_2.

[11] Swanson SA, Crow SJ, Le Grange D, Swendsen J, Merikangas KR. Prevalence and correlates of eating disorders in adolescents: results from the national comorbidity survey replication adolescent supplement. Arch Gen Psychiatry. 2011;68(7):714–23. 21383252 10.1001/archgenpsychiatry.2011.22 PMC5546800

[12] Arcelus J, Mitchell AJ, Wales J, Nielsen S. Mortality rates in patients with anorexia nervosa and other eating disorders: a meta-analysis of 36 studies. Arch Gen Psychiatry. 2011;68(7):724–31. 21727255 10.1001/archgenpsychiatry.2011.74

[13] Steinhausen H-C. The outcome of anorexia nervosa in the 20th century. Am J Psychiatry. 2002;159(8):1284–93. 12153817 10.1176/appi.ajp.159.8.1284

[14] Holland LA, Bodell LP, Keel PK. Psychological factors predict eating disorder onset and maintenance at 10-year follow-up. Eur Eat Disord Rev. 2013;21(5):405–10. 23847146 10.1002/erv.2241 PMC4096787

[15] Steinglass JE, Walsh BT. Neurobiological model of the persistence of anorexia nervosa. J Eating Disord. 2016;4(1):19. 10.1186/s40337-016-0106-2 PMC4870737 27195123

[16] Gama CS, Kunz M, Magalhães PVS, Kapczinski F. Staging and neuroprogression in bipolar disorder: a systematic review of the literature. Rev Bras Psiquiatr. 2013;35(1):70–4. 23567604 10.1016/j.rbp.2012.09.001

[17] Moylan S, Maes M, Wray N, Berk M. The neuroprogressive nature of major depressive disorder: pathways to disease evolution and resistance, and therapeutic implications. Mol Psychiatry. 2013;18(5):595–606. 22525486 10.1038/mp.2012.33

[18] O’Hara CB, Campbell IC, Schmidt U. A reward-centred model of anorexia nervosa: A focussed narrative review of the neurological and psychophysiological literature. Neurosci Biobehav Rev. 2015;52:131–52. 25735957 10.1016/j.neubiorev.2015.02.012

[19] Treasure J, Stein D, Maguire S. Has the time come for a staging model to map the course of eating disorders from high risk to severe enduring illness? An examination of the evidence. Early Interv Psychiatry. 2015;9(3):173–84. 25263388 10.1111/eip.12170

[20] Austin A, Flynn M, Richards K, Hodsoll J, Duarte TA, Robinson P, et al. Duration of untreated eating disorder and relationship to outcomes: a systematic review of the literature. Eur Eat Disord Rev. 2021;29(3):329–45. 32578311 10.1002/erv.2745

[21] Beat. Three-and-a-half-year delay for eating disorder treatment worsens illness and cost to the NHS. 2020. Available from: https://www.beateatingdisorders.org.uk/news/beat-news/three-half-year-delay-eating-disorder-treatment/.

[22] Weigel A, Rossi M, Wendt H, Neubauer K, von Rad K, Daubmann A, et al. Duration of untreated illness and predictors of late treatment initiation in anorexia nervosa. J Public Health. 2014;22(6):519–27.

[23] Potterton R, Austin A, Allen K, Lawrence V, Schmidt U. “I’m not a teenager, I’m 22. Why can’t I snap out of it?”: a qualitative exploration of seeking help for a first-episode eating disorder during emerging adulthood. J Eating Disord. 2020;8(1):1–14. 10.1186/s40337-020-00320-5 PMC7469268 32905371

[24] Potterton R, Richards K, Allen K, Schmidt U. Eating disorders during emerging adulthood: a systematic scoping review. Front Psychol. 2019;10:3062. 32082210 10.3389/fpsyg.2019.03062 PMC7005676

[25] Bryant E, Spielman K, Le A, Marks P, Aouad P, Barakat S, et al. Screening, assessment and diagnosis in the eating disorders: findings from a rapid review. J Eat Disord. 2022;10(1):78. 35672777 10.1186/s40337-022-00597-8 PMC9175461

[26] Coffino JA, Udo T, Grilo CM. Rates of help-seeking in US adults with lifetime DSM-5 eating disorders: prevalence across diagnoses and differences by sex and ethnicity/race. Mayo Clin Proc. 2019;94(8):1415–26. 31324401 10.1016/j.mayocp.2019.02.030 PMC6706865

[27] Hamilton A, Mitchison D, Basten C, Byrne S, Goldstein M, Hay P, et al. Understanding treatment delay: perceived barriers preventing treatment-seeking for eating disorders. Aust N Z J Psychiatry. 2022;56(3):248–59. 34250844 10.1177/00048674211020102

[28] Connor C, Birchwood M, Freemantle N, Palmer C, Channa S, Barker C, et al. Don’t turn your back on the symptoms of psychosis: the results of a proof-of-principle, quasi-experimental intervention to reduce duration of untreated psychosis. BMC Psychiatry. 2016;16(1):127. 27145865 10.1186/s12888-016-0816-7 PMC4855493

[29] Schmidt U, Brown A, McClelland J, Glennon D, Mountford VA. Will a comprehensive, person-centered, team-based early intervention approach to first episode illness improve outcomes in eating disorders? Int J Eat Disord. 2016;49(4):374–7. 27084796 10.1002/eat.22519

[30] Brown A, McClelland J, Boysen E, Mountford V, Glennon D, Schmidt U. The FREED Project (first episode and rapid early intervention in eating disorders): service model, feasibility and acceptability. Early Interv Psychiatry. 2016;12(2):250–7. 27619198 10.1111/eip.12382

[31] McClelland J, Hodsoll J, Brown A, Lang K, Boysen E, Flynn M, et al. A pilot evaluation of a novel First Episode and Rapid Early Intervention service for Eating Disorders (FREED). Eur Eat Disord Rev. 2018;26(2):129–40. 29460477 10.1002/erv.2579

[32] Flynn M, Austin A, Lang K, Allen K, Bassi R, Brady G, et al. Assessing the impact of first episode rapid early intervention for eating disorders on duration of untreated eating disorder: a multi-centre quasi-experimental study. Eur Eat Disord Rev. 2021;29(3):458–71. 33112472 10.1002/erv.2797

[33] Fukutomi A, Austin A, McClelland J, Brown A, Glennon D, Mountford V, et al. First episode rapid early intervention for eating disorders: a two-year follow-up. Early Interv Psychiatry. 2020;14(1):137–41. 31617325 10.1111/eip.12881

[34] Richards KL, Hyam L, Allen KL, Glennon D, Di Clemente G, Semple A, et al. National roll-out of early intervention for eating disorders: process and clinical outcomes from first episode rapid early intervention for eating disorders. Early Interv Psychiatry. 2023;17(2):202–211. 10.1111/eip.13317 35676870

[35] The AHSN Network. Early Intervention Eating Disorders: Supporting mental health teams across England to speed up diagnosis and treatment of eating disorders in young people 2020. Available from: https://www.ahsnnetwork.com/about-academic-health-science-networks/national-programmes-priorities/early-intervention-eating-disorders.

[36] Waller G, Schmidt U, Treasure J, Murray K, Aleyna J, Emanuelli F, et al. Problems across care pathways in specialist adult eating disorder services. Psychiatr Bull. 2009;33(1):26–9.

[37] Jenkins PE. Reducing non-attendance rates for assessment at an eating disorders service: a quality improvement initiative. Community Ment Health J. 2017;53(7):878–82. 28185137 10.1007/s10597-017-0118-7

[38] Muir S, Newell C, Griffiths J, Walker K, Hooper H, Thomas S, et al. MotivATE: a pretreatment web-based program to improve attendance at UK outpatient services among adults with eating disorders. JMIR Research Protocols. 2017;6(7):e146. 28747295 10.2196/resprot.7440 PMC5550733

[39] Gunnarsdottir GM, Palsson SP, Thorsteinsdottir G. Eating Disorder Treatment in Iceland-Treatment adherence, psychiatric co-morbidities and factors influencing drop-out. Laeknabladid. 2015;101(5):251–7. 26019127 10.17992/lbl.2015.05.26

[40] Innes NT, Clough BA, Casey LM. Assessing treatment barriers in eating disorders: A systematic review. Eat Disord. 2017;25(1):1–21. 27485375 10.1080/10640266.2016.1207455

[41] Regan P, Cachelin FM, Minnick AM. Initial treatment seeking from professional health care providers for eating disorders: a review and synthesis of potential barriers to and facilitators of “first contact.” Int J Eat Disord. 2017;50(3):190–209. 28134980 10.1002/eat.22683

[42] Ali K, Fassnacht DB, Farrer L, Rieger E, Feldhege J, Moessner M, et al. What prevents young adults from seeking help? Barriers toward help-seeking for eating disorder symptomatology. Int J Eat Disord. 2020;53(6):894–906. 32239776 10.1002/eat.23266

[43] Gratwick-Sarll K, Bentley C, Harrison C, Mond J. Poor self-recognition of disordered eating among girls with bulimic-type eating disorders: cause for concern? Early Interv Psychiatry. 2016;10(4):316–23. 25112818 10.1111/eip.12168

[44] Vitousek K, Watson S, Wilson GT. Enhancing motivation for change in treatment-resistant eating disorders. Clin Psychol Rev. 1998;18(4):391–420. 9638355 10.1016/s0272-7358(98)00012-9

[45] Michie S, van Stralen MM, West R. The behaviour change wheel: a new method for characterising and designing behaviour change interventions. Implementation Science. 2011;6(1):42. 10.1186/1748-5908-6-42. 10.1186/1748-5908-6-42 PMC3096582 21513547

[46] Atkins L, Michie S. Designing interventions to change eating behaviours. Proceedings of the Nutritional Society. 2015;74(2):164–70. 10.1017/S0029665115000075 25998679

[47] Kazdin AE, Fitzsimmons-Craft EE, Wilfley DE. Addressing critical gaps in the treatment of eating disorders. Int J Eat Disord. 2017;50(3):170–89. 28102908 10.1002/eat.22670 PMC6169314

[48] Crisafulli MA, Von Holle A, Bulik CM. Attitudes towards anorexia nervosa: the impact of framing on blame and stigma. Int J Eat Disord. 2008;41(4):333–9. 18186057 10.1002/eat.20507

[49] Lebowitz MS, Woo-kyoung A, Nolen-Hoeksema S. Fixable or fate? Perceptions of the biology of depression. J Consult Clin Psychol. 2013;81(3):518–27. 23379262 10.1037/a0031730 PMC3958946

[50] Lebowitz MS, Woo-kyoung A. Emphasizing Malleability in the biology of depression: Durable effects on perceived agency and prognostic pessimism. Behav Res Ther. 2015;71:125–30. 26112398 10.1016/j.brat.2015.06.005 PMC4501891

[51] Kemp JJ, Lickel JJ, Deacon BJ. Effects of a chemical imbalance causal explanation on individuals’ perceptions of their depressive symptoms. Behav Res Ther. 2014;56:47–52. 24657311 10.1016/j.brat.2014.02.009

[52] Lebowitz MS, Pyun JJ, Woo-kyoung A. Biological explanations of generalized anxiety disorder: effects on beliefs about prognosis and responsibility. Psychiatr Serv. 2014;65(4):498–503. 24337358 10.1176/appi.ps.201300011

[53] Lebowitz MS, Rosenthal JE, Woo-kyoung A. Effects of biological versus psychosocial explanations on stigmatization of children with ADHD. J Atten Disord. 2016;20(3):240–50. 23264369 10.1177/1087054712469255

[54] Farrell NR, Lee AA, Deacon BJ. Biological or psychological? Effects of eating disorder psychoeducation on self-blame and recovery expectations among symptomatic individuals. Behav Res Ther. 2015;74:32–7. 26378721 10.1016/j.brat.2015.08.011

[55] Ogston-Tuck S, Baume K, Clarke C, Heng S. Understanding the patient experience through the power of film: a mixed method qualitative research study. Nurse Educ Today. 2016;46:69–74. 27607526 10.1016/j.nedt.2016.08.025

[56] Bol N, Smets EMA, Rutgers MM, Burgers JA, de Haes HCJM, Loos EF, et al. Do videos improve website satisfaction and recall of online cancer-related information in older lung cancer patients? Patient Educ Couns. 2013;92(3):404–12. 23820196 10.1016/j.pec.2013.06.004

[57] Meppelink CS, van Weert JCM, Brosius A, Smit EG. Dutch health websites and their ability to inform people with low health literacy. Patient Educ Couns. 2017;100(11):2012–9. 28624261 10.1016/j.pec.2017.06.012

[58] Krishnan S, Dalvie S. From unwanted pregnancy to safe abortion: Sharing information about abortion in Asia through animation. Reprod Health Matters. 2015;23(45):126–35. 26278840 10.1016/j.rhm.2015.06.013

[59] Szeszak S, Man R, Love A, Langmack G, Wharrad H, Dineen RA. Animated educational video to prepare children for MRI without sedation: evaluation of the appeal and value. Pediatr Radiol. 2016;46(12):1744–50. 27568023 10.1007/s00247-016-3661-4

[60] Davies K, Armitage CJ, Lin Y-L, Munro J, Walsh T, Callery P. Development of an implementation intention-based intervention to change children’s and parent-carers’ behaviour. Pilot and Feasibility Studies. 2017;4(1):20. 10.1186/s40814-017-0171-6. 10.1186/s40814-017-0171-6 PMC5513026 28725453

[61] March S, Day J, Zieschank K, Ireland M. The interactive child distress screener: development and preliminary feasibility testing. JMIR Mhealth Uhealth. 2018;6(4):e90. 29674310 10.2196/mhealth.9456 PMC5934532

[62] Hatfield KL, Hinck A, Birkholt MJ. Seeing the visual in argumentation: a rhetorical analysis of Unicef Belgium’s Smurf Public Service Announcement. Argumentation and Advocacy. 2007;43(3–4):144–51.

[63] Musiat P, Hoffmann L, Schmidt U. Personalised computerised feedback in E-mental health. J Ment Health. 2012;21(4):346–54. 22315961 10.3109/09638237.2011.648347

[64] Sapyta J, Riemer M, Bickman L. Feedback to clinicians: theory, research, and practice. J Clin Psychol. 2005;61(2):145–53. 15609360 10.1002/jclp.20107

[65] Knaup C, Koesters M, Schoefer D, Becker T, Puschner B. Effect of feedback of treatment outcome in specialist mental healthcare: meta-analysis. Br J Psychiatry. 2009;195(1):15–22. 19567889 10.1192/bjp.bp.108.053967

[66] Lopez C, Roberts ME, Tchanturia K, Treasure J. Using neuropsychological feedback therapeutically in treatment for anorexia nervosa: two illustrative case reports. Eur Eat Disord Rev. 2008;16(6):411–20. 18288783 10.1002/erv.866

[67] Moessner M, Minarik C, Özer F, Bauer S. Can an internet-based program for the prevention and early intervention in eating disorders facilitate access to conventional professional healthcare? J Ment Health. 2016;25(5):441–7. 26850624 10.3109/09638237.2016.1139064

[68] Schmidt U, Landau S, Pombo-Carril MG, Bara-Carril N, Reid Y, Murray K, et al. Does personalized feedback improve the outcome of cognitive-behavioural guided self-care in bulimia nervosa? A preliminary randomized controlled trial. Br J Clin Psychol. 2006;45(1):111–21. 16480570 10.1348/014466505X29143

[69] Davidsen AH, Poulsen S, Waaddegaard M, Lindschou J, Lau M. Feedback versus no feedback in improving patient outcome in group psychotherapy for eating disorders (F-EAT): protocol for a randomized clinical trial. Trials. 2014;15(1):138. 10.1186/1745-6215-15-138. 10.1186/1745-6215-15-138 PMC4005398 24754974

[70] Bauer S, Okon E, Meermann R, Kordy H. Technology-enhanced maintenance of treatment gains in eating disorders: efficacy of an intervention delivered via text messaging. J Consult Clin Psychol. 2012;80(4):700–6. 22545736 10.1037/a0028030

[71] Shapiro JR, Bauer S, Andrews E, Pisetsky E, Bulik-Sullivan B, Hamer RM, et al. Mobile therapy: Use of text-messaging in the treatment of bulimia nervosa. Int J Eat Disord. 2010;43(6):513–9. 19718672 10.1002/eat.20744

[72] Robinson S, Perkins S, Bauer S, Hammond N, Treasure J, Schmidt U. Aftercare intervention through text messaging in the treatment of bulimia nervosa—feasibility pilot. Int J Eat Disord. 2006;39(8):633–8. 16937381 10.1002/eat.20272

[73] Chan A-W, Tetzlaff JM, Altman DG, Laupacis A, Gøtzsche PC, Krleža-Jerić K, et al. SPIRIT 2013 statement: defining standard protocol items for clinical trials. Ann Intern Med. 2013;158(3):200–7. 23295957 10.7326/0003-4819-158-3-201302050-00583 PMC5114123

[74] Thabane L, Lancaster G. A guide to the reporting of protocols of pilot and feasibility trials. Pilot Feasibility Stud. 2019;5:37. 30858987 10.1186/s40814-019-0423-8 PMC6393983

[75] Hill LS, Reid F, Morgan JF, Lacey JH. SCOFF, the development of an eating disorder screening questionnaire. Int J Eat Disord. 2010;43(4):344–51. 19343793 10.1002/eat.20679

[76] Teare MD, Dimairo M, Shephard N, Hayman A, Whitehead A, Walters SJ. Sample size requirements to estimate key design parameters from external pilot randomised controlled trials: a simulation study. Trials. 2014;15(1):264. 10.1186/1745-6215-15-264. 10.1186/1745-6215-15-264 PMC4227298 24993581

[77] Cavazos-Rehg P, Min C, Fitzsimmons-Craft EE, Savoy B, Kaiser N, Riordan R, et al. Parental consent: a potential barrier for underage teens' participation in an mHealth mental health intervention. Internet Interv. 2020;21:100328. 10.1016/j.invent.2020.100328 PMC7276447 32528858

[78] Stice E, Telch CL, Rizvi SL. Development and validation of the Eating Disorder Diagnostic Scale: a brief self-report measure of anorexia, bulimia, and binge-eating disorder. Psychol Assess. 2000;12(2):123–32. 10887758 10.1037//1040-3590.12.2.123

[79] Rollnick S, Mason P, Butler C. Health behavior change: a guide for practitioners. Edinburgh, Scotland: Churchill Livingston; 1999.

[80] Kroenke K, Spitzer RL. The PHQ-9: a new depression diagnostic and severity measure. Psychiatr Ann. 2002;32(9):509–15.

[81] Spitzer RL, Kroenke K, Williams JB, Löwe B. A brief measure for assessing generalized anxiety disorder: the GAD-7. Arch Intern Med. 2006;166(10):1092–7. 16717171 10.1001/archinte.166.10.1092

[82] Vogel DL, Wester SR. To seek help or not to seek help: the risks of self-disclosure. J Couns Psychol. 2003;50(3):351.

[83] McLean SA, Paxton SJ, Wertheim EH, Masters J. Photoshopping the selfie: Self photo editing and photo investment are associated with body dissatisfaction in adolescent girls. Int J Eat Disord. 2015;48(8):1132–40. 26311205 10.1002/eat.22449

[84] Rodgers RF, McLean SA, Gordon CS, Slater A, Marques MD, Jarman HK, et al. Development and Validation of the Motivations for Social Media Use Scale (MSMU) among adolescents. Adolesc Res Rev. 2021;6(4):425–35.

[85] Jassi A, Lenhard F, Krebs G, Gumpert M, Jolstedt M, Andrén P, et al. The work and social adjustment scale, youth and parent versions: psychometric evaluation of a brief measure of functional impairment in young people. Child Psychiatry Hum Dev. 2020;51(3):453–60. 32006302 10.1007/s10578-020-00956-z PMC7235060

[86] Herdman M, Gudex C, Lloyd A, Janssen M, Kind P, Parkin D, et al. Development and preliminary testing of the new five-level version of EQ-5D (EQ-5D-5L). Qual Life Res. 2011;20(10):1727–36. 21479777 10.1007/s11136-011-9903-x PMC3220807

[87] van Reenen M, Janssen B, Oppe M, Kreimeier S, Greiner W. EQ-5D-Y User Guide Version 1.0. 2014.

[88] Perez J, Jin H, Russo DA, Stochl J, Painter M, Shelley G, et al. Clinical effectiveness and cost-effectiveness of tailored intensive liaison between primary and secondary care to identify individuals at risk of a first psychotic illness (the LEGs study): a cluster-randomised controlled trial. Lancet Psychiatry. 2015;2(11):984–93. 26296562 10.1016/S2215-0366(15)00157-1 PMC4641188

[89] Gowers S, Clark A, Roberts C, Byford S, Barrett B, Griffiths A, et al. A randomised controlled multicentre trial of treatments for adolescent anorexia nervosa including assessment of cost-effectiveness and patient acceptability-the TOuCAN trial. Health Technol Assess. 2010;14(15):1–98. 20334748 10.3310/hta14150

[90] Schmidt U, Sharpe H, Bartholdy S, Bonin E-M, Davies H, Easter A, et al. Treatment of anorexia nervosa: a multimethod investigation translating experimental neuroscience into clinical practice. Programme Grants for Applied Research. 2017;5(16). 10.3310/pgfar05160. 28872815

[91] World Health Organization. Monitoring and evaluating digital health interventions: a practical guide to conducting research and assessment. Geneva. 2016. Available from: http://apps.who.int/iris/bitstream/handle/10665/252183/9789241511766-eng.pdf;jsessionid=FCE997C408672469A4896FB66C955999?sequence=1.

[92] Michie S, Yardley L, West R, Patrick K, Greaves F. Developing and evaluating digital interventions to promote behavior change in health and health care: recommendations resulting from an international workshop. J Med Internet Res. 2017;19(6):e232. 28663162 10.2196/jmir.7126 PMC5509948

[93] Saunders B, Sim J, Kingstone T, Baker S, Waterfield J, Bartlam B, et al. Saturation in qualitative research: exploring its conceptualization and operationalization. Qual Quant. 2018;52(4):1893–907. 29937585 10.1007/s11135-017-0574-8 PMC5993836

[94] Ritchie J, Spencer L. Qualitative data analysis for applied policy research. 2022/12/19. In: Analysing qualitative data. London: Routledge; 1994. p. 173–94.

[95] Braun V, Clarke V. Thematic Analysis: A Practical Guide. London: SAGE Publications LTd; 2022.

[96] Braun V, Clarke V. Using thematic analysis in psychology. Qual Res Psychol. 2006;3(2):77–101.

[97] INVOLVE. Payment and reimbursement rates for public involvement National Institute for Health Research; 2009. Available from: https://www.invo.org.uk/wp-content/uploads/2011/12/NIHRProgrammesPaymentRates2009.pdf.

[98] Eldridge SM, Chan CL, Campbell MJ, Bond CM, Hopewell S, Thabane L, et al. CONSORT 2010 statement: extension to randomised pilot and feasibility trials. BMJ. 2016;355:i5239. 27777223 10.1136/bmj.i5239 PMC5076380

