# Supplementary material for: Shortening duration of untreated illness in young people with first episode eating disorders: protocol of a randomised controlled feasibility trial of a smartphone friendly multi-modal decision-making tool (FREED-M) to improve help-seeking
Source: Pilot Feasibility Stud. 2025 Feb 7;11:14. doi: 10.1186/s40814-024-01585-2 (PMC11804029; doi:10.1186/s40814-024-01585-2)
Supplement: Supplementary file 1 — Supplementary Material 1. [file 40814_2024_1585_MOESM1_ESM.docx]

Appendices

Appendix 1: Topic Guide for Qualitative Interviews:

***Both groups:***

1. What were your hopes and expectations of the FREED-M study before signing up?

- Prompts (if needed)
  - What were you hoping to get out of FREED-M?
  - How did you hear about us? (if not detailed in the survey)
  - What made you want to get involved?
  - Did you have any concerns at the start, or was there anything that put you off?

1. We asked you to complete quite a lot of questionnaires at the beginning of the study, at 1 month and at 3 months. What were your thoughts about these?

- Prompts (if needed):
  - How relevant did the questions seem?
  - Were they difficult or easy to answer? Thought-provoking? Encouraging reflection?
  - How did you find the amount of questions you had to answer?
    - Did that feel okay, or was it too much?

1. *Intervention group:* What changes in yourself, if any, did you notice after starting to use the FREED-M tool?

*Control group:* What changes in yourself, if any, did you notice after joining the FREED-M study?

- Prompts for both groups (if needed)
  - Have you learned anything new?
  - Have you noticed any changes in how you feel about getting support for your eating?
  - Were there any aspects that made you feel a bit better or made you feel worse?

1. How did you find the FREED-M [tool/study]?

- Prompts (if needed)
  - Did it meet your expectations?
  - Was the content what you were expecting?
  - Were there any things you expected to be included but weren’t?

***Intervention arm only:***

1. What did you think about the videos?

- Prompts (if needed): help-seeking, the gut, social media, EDs & the brain.
  - Were there any topics in the videos which you found particularly helpful or unhelpful?
    - Why?
  - Were the videos relevant to you?
  - What aspect of the videos have stuck with you?
  - Were there any parts which didn’t feel so helpful? (if not covered above)
  - How did you find the animations?

1. After each video you were presented with additional resources. Did you access any of those resources?

- Prompts (if needed): help-seeking, the gut, social media, EDs & the brain.
  - How did you feel about the resources?
  - Is there something that would make you look into it?
  - Were the resources feel relevant to you?
  - Were there any aspects of the resources which you found particularly helpful or less helpful?

1. You’ll remember that the tool gives you some feedback about your responses to the surveys. What did you think about this feedback?
   - Prompts (if needed)
     - What do you remember about the feedback?
     - Was it given at the right time?
     - Did the way it was written make sense to you?
     - Was the feedback relevant to you?
     - Was it the right amount of feedback – would you have preferred more or less?
2. Do you think FREED-M tool could help other young people? if so, how?
   - Prompts (if needed)
     - Who would this be most helpful for?
     - Right audience of 16-25 yo?

***Control arm only:***

5) What did you think about the Beat website?

- Prompts (if needed)
  - How often did you access it?
  - Were the resources relevant to you?
  - What did you find helpful or unhelpful?

6) After the study ended, you were given access to the FREED-M tool. Did you take a look at the materials?

7) *If yes to question 6:* How did you find the FREED-M tool?

- Prompts (if needed)
  - Did it meet your expectations?
  - Was the content what you were expecting?
  - Were there any things you expected to be included but weren’t?

*If no to question 6:* What prevented you from looking or made you decide not to look at the FREED-M tool?

- Prompts (if needed)
  - How relevant did the tool sound to you?
  - Do you think it would be helpful or unhelpful for you?
  - Was there anything that could have made the tool sound more appealing to you?

8) *If yes to Q6:* Do you think it could help other young people? If so, how?

- - Prompts (if needed)
    - Who would this be most helpful for?

*If no to Q6:* Based on what you know about the FREED-M tool, do you think it could help other young people? If so, how?

- - Prompts (if needed)
    - Who would this be most helpful for?

***Both groups:***

1. Do you think there was anything missing from the FREED-M [tool/study]?

- Prompts (if needed): topics, information, explanations

1. What did you think of the design/look of the FREED-M [tool/study]?
2. How easy/difficult did you find it to use the FREED-M [tool/study]? Technical difficulties

- Mobile or computer?

1. How engaging did you find the FREED-M [tool/study]?

- Prompts for *intervention group* (if needed):
  - Did you find it easy to integrate into your weekly routine?
  - Were there any times when it was annoying or got in the way?
  - What would have encouraged you to use it more?
- Prompts for *control group* (if needed):
  - Did you find it easy to respond to the weekly surveys?
  - Were there any times when it was annoying or got in the way?
  - What would have encouraged you to respond to the surveys more consistently?

Health economics questions:

*Now I’m going to ask you a few specific questions about two questionnaires you have already completed as part of the study, to help us make sure that we are using the best questionnaires for a larger trial. The first questionnaire I would like to talk about is a questionnaire that is designed to capture your quality of life as it relates to your health. Would you be able to have a read through it and let me know when you’re done? [Wait until the participants reads through the questionnaire.]*

1. Did you find this questionnaire easy to complete?
2. Did you find the questions relevant to you?

- Prompts (if needed):
  - Are there any areas missing that would be relevant to young people with eating disorders?

1. Were there any questions in this questionnaire that you did not understand or thought could have been clearer?
   - If yes, which questions?

*The second questionnaire I would like to talk about asks about the healthcare services that you have used in the past 3 months. Would you be able to have a read through it and let me know when you’re done? [Wait until the participants reads through the questionnaire.]*

1. Were there any questions in this questionnaire that you did not understand or thought could have been clearer?
   - If yes, which questions?
   - Are wee missing nay components

Conclusion:

1. Is there anything else would you like to tell us about that we haven’t asked about yet, or other ways that you think the FREED-M [tool/study] could be improved?
